# Saliva antibody-fingerprint of reactivated latent viruses after mild/asymptomatic COVID-19 is unique in patients with myalgic-encephalomyelitis/chronic fatigue syndrome

**DOI:** 10.3389/fimmu.2022.949787

**Published:** 2022-10-20

**Authors:** Eirini Apostolou, Muhammad Rizwan, Petros Moustardas, Per Sjögren, Bo Christer Bertilson, Björn Bragée, Olli Polo, Anders Rosén

**Affiliations:** ^1^ Division of Cell Biology, Department of Biomedical and Clinical Sciences, Linköping University, Linköping, Sweden; ^2^ Division of Family Medicine and Primary Care, Department of Neurobiology, Care Sciences and Society, Karolinska Institute, Stockholm, Sweden; ^3^ ME-center, Bragée Clinics, Stockholm, Sweden

**Keywords:** COVID-19, latent virus, herpesvirus reactivation, myalgic encephalomyelitis/chronic fatigue syndrome, ME/CFS, EBV, HERV, HHV6A

## Abstract

**Background:**

Myalgic encephalomyelitis/chronic fatigue syndrome (ME/CFS) is a chronic disease considered to be triggered by viral infections in a majority of cases. Symptoms overlap largely with those of post-acute sequelae of COVID-19/long-COVID implying common pathogenetic mechanisms. SARS-CoV-2 infection is risk factor for sustained latent virus reactivation that may account for the symptoms of post-viral fatigue syndromes. The aim of this study was first to investigate whether patients with ME/CFS and healthy donors (HDs) differed in their antibody response to mild/asymptomatic SARS-CoV-2 infection. Secondly, to analyze whether COVID-19 imposes latent virus reactivation in the cohorts.

**Methods:**

Anti-SARS-CoV-2 antibodies were analyzed in plasma and saliva from non-vaccinated ME/CFS (n=95) and HDs (n=110) using soluble multiplex immunoassay. Reactivation of human herpesviruses 1-6 (HSV1, HSV2, VZV, EBV, CMV, HHV6), and human endogenous retrovirus K (HERV-K) was detected by anti-viral antibody fingerprints in saliva.

**Results:**

At 3-6 months after mild/asymptomatic SARS-CoV-2 infection, virus-specific antibodies in saliva were substantially induced signifying a strong reactivation of latent viruses (EBV, HHV6 and HERV-K) in both cohorts. In patients with ME/CFS, antibody responses were significantly stronger, in particular EBV-encoded nuclear antigen-1 (EBNA1) IgG were elevated in patients with ME/CFS, but not in HDs. EBV-VCA IgG was also elevated at baseline prior to SARS-infection in patients compared to HDs.

**Conclusion:**

Our results denote an altered and chronically aroused anti-viral profile against latent viruses in ME/CFS. SARS-CoV-2 infection even in its mild/asymptomatic form is a potent trigger for reactivation of latent herpesviruses (EBV, HHV6) and endogenous retroviruses (HERV-K), as detected by antibody fingerprints locally in the oral mucosa (saliva samples). This has not been shown before because the antibody elevation is not detected systemically in the circulation/plasma.

## Introduction

Myalgic encephalomyelitis/chronic fatigue syndrome (ME/CFS) is a heterogeneous, chronic, and disabling morbidity with a unknown pathogenesis and etiology that manifests with a range of symptoms such as post-exertional malaise (PEM), postural orthostatic tachycardia syndrome (POTS), brain fog, cognitive impairment, unrefreshing sleep, myalgia and headache (1, 2). In the majority, although not in all cases, the onset occurs following a viral or bacterial infection (3, 4), with symptoms persisting and patient health deteriorating even after the resolution of the initial infection. Particularly, ME/CFS is mainly triggered by severe infections, such as Epstein-Barr virus (EBV)-induced infectious mononucleosis, *Coxiella burnetii* (Q-fever), Ebola virus (Ebola hemorrhagic fever), or SARS-CoV virus (post-SARS syndrome) (5–7). Clusters of conditions resembling ME/CFS have been documented following epidemic infectious outbreaks (8).

The symptoms of post-infectious fatigue syndromes and specifically ME/CFS are similar to those of post-acute sequelae of COVID-19 (PASC, also named long-COVID), that occurs in about 30% of infected individuals independently of severity of SARS-CoV-2 infection (9). Viral infections may have both immediate and long-term complications. During the acute infection phase, certain viruses invade their target cells and hijack to their own advantage the cellular machinery including the mitochondria, as observed for SARS-CoV-2 (10), EBV (11) and HHV6 (12). The ensuing compromised cellular energy production, may affect a wide range of cellular functions, and trigger prolonged immune and autoimmune responses (9). Proposed disease-models for ME/CFS include chronic infection, chronic inflammation, autoimmunity, impaired energy metabolism, dysfunction of the autonomic nervous system, and/or hormonal dysregulation (6). However, none of the models explain comprehensively the clinical picture and the long-term health deterioration occurring in ME/CFS after the triggering infection event.

Reactivation of latent viruses occurs frequently in healthy individuals upon physical or mental stress or traumatic events. However, this is balanced by the counteractive action of a functional immune system. SARS-CoV-2 infection is a potential risk factor for sustained latent virus reactivation (13–15). So far, studies have reported sustained latent virus reactivation in cases of severe SARS-CoV-2 infection in hospitalized/intensive care unit (ICU) treated patients, which pose a severe threat to the patient’s life. Serological analysis in patients with critical COVID-19 confirmed the reactivation of herpesviruses by demonstrating increased IgG antibody levels against human simplex 1 (HSV1) (16), varicella zoster virus (VZV), EBV, and cytomegalovirus (CMV), as well as detectable EBV and CMV viremia in blood (17). In ICU-admitted SARS-CoV-2 patients, HSV1, VZV, EBV, CMV, and human herpesvirus 6 (HHV6) has been reported to be reactivated (18). EBV reactivation specifically has been associated with delayed recovery, and thus proposed as an underlying cause of PASC (19), whereas similar post-viral fatigue syndromes have been reported for HSV and CMV (16). Apart from symbiotic herpesviruses that are acquired early in life, SARS-CoV-2 infection has been reported to upregulate the expression of specific human endogenous retroviruses (HERVs) both in bronchoalveolar lavage fluid cells and in peripheral blood mononuclear cells (PBMC) (20). HERVs are unique endogenous retroelements that have been acquired during human evolution and represent a substantial proportion (8%) of the human genome. Although HERVs are replication deficient, the transcription of endogenous retroelements is evident. HERVs are responsive to both cell-intrinsic and external signals, including viral infections like SARS-CoV-2 (21, 22).

In patients with ME/CFS, the involvement of latent viruses in the initiation and perpetuation of the disease is intensively investigated but difficult to address. High rate of active EBV infection has been reported, suggesting that at least in a subset of patients, EBV is important factor for the development of the disease (23). However, reports on no correlation to herpesvirus infection highlight that the issue is not yet clear (24, 25) Additionally, viral loads for HHV6B and HHV7, were previously reported to be higher in saliva samples of patients with ME/CFS compared to healthy controls (26), whereas partial HHV6 reactivation has been demonstrated (HHV6 small noncoding RNA U14 in whole blood) in 40% of the patients (12).

Both biological and clinical markers point towards a state of acquired immunosuppression in severe SARS-CoV-2 infection that may explain the non-supervised, prolonged latent viral reactivation in these patients (27, 28). However, most infected persons show mild or no symptoms (29) and reports on long-COVID cases do not necessarily correlate with a severe initial infection (19). The objective of this study was to investigate anti-viral immune responses against (re)activated ubiquitous herpesviruses and endogenous retroviruses after mild or asymptomatic SARS-CoV-2 infection in patients with ME/CFS and matched healthy donors.

## Results

### Saliva and plasma antibody response against SARS/CoV-2 in patients with ME/CFS and healthy donors

**Figure 1:**
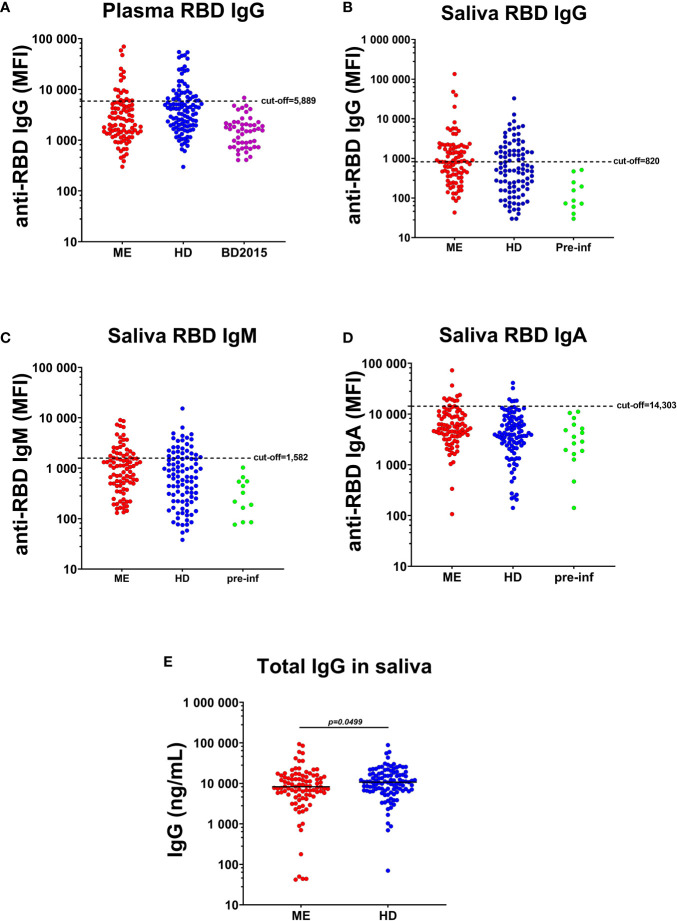
Plasma and saliva antibody responses against SARS-CoV-2-RBD (RBD). **(A)** IgG in plasma of patients with ME/CFS (ME), healthy donors (HDs) and blood donors collected before COVID-19, during 2015 (BD2015). Antibody responses against RBD in the saliva of ME, HD and a group of study participants seroconverted during the course of the study (pre-infection donors, pre-inf), for **(B)** IgG, **(C)** IgM and **(D)** IgA class. Cut-off threshold levels used to define SARS-CoV-2 positive/negative subgroups are indicated with dashed horizontal lines. For IgG responses in plasma, cut-off level was calculated from BD2015 IgG levels (BD2015, n=50; mean MFI + 3SD = 5889 MFI). For antibody responses in the saliva, cut-off levels were calculated from antibody titers of pre-inf donors (n=19; mean MFI + 3SD = 820 MFI for IgG, 1582 MFI for IgM and 14303 for IgA). **(E)** Concentration of total IgG (ng/mL) in the saliva of patients with ME (n=95) and HDs (n=110). Lines represents mean MFI (median fluorescence index). Statistically significant difference was calculated by nonparametric Wilcoxon test.

Antibody responses against SARS-CoV-2-spike protein receptor-binding domain (RBD) were analyzed in plasma and saliva of all participant before vaccination (**Figure 1**). COVID-19 participants in this study experienced mild or no symptoms and did not require hospitalization. At the time of sampling, 3-6 month after the start of the pandemic, 18/95 (19%) of the ME/CFS cohort were plasma RBD IgG-positive vs. 35/110 (32%) of the HDs (**Figure 1**, mean MFI 19,678 vs 18,071, *p*=*0.94*). Plasma samples collected in 2015 from healthy blood donors (BD2015, n=50) were used to define the cut-off levels (mean MFI+3SD; 5,889 MFI) (**Figure 1A**). This relatively high cut-off level can be explained by multiple binding-sites on the spike protein used in the multiplex assay, and the presence of low-affinity IgG in non-infected pre-pandemic (2015 blood donor) plasma.

Similar antibody levels in adult non-infected persons were also observed by Dobano et al. (30). RBD antibodies of IgG, IgM, IgA classes, were released locally onto the oral mucosa, as detected by antibody ‘fingerprinting’ in saliva in both cohorts: 42/95 (44%) of ME/CFS donors were RBD IgG^+^ vs. 28/110 (25%) of HDs **(** **Figure 1B** **).** Cut-off levels for saliva RBD antibodies were estimated from 19 participants (15 HD and 4 ME/CFS; mean MFI+3SD), who were RBD IgG negative in plasma, and RBD IgG, IgM, and IgA negative in saliva, at the time of study inclusion and then got infected during the course of this study with documented positive PCR result and/or established COVID-19 symptoms, as well as positive RBD-antibodies after infection in saliva.

Taken together with RBD IgM and RBD IgA in saliva (**Figures 1C, D**), 58% of ME/CFS and 41% of HDs had saliva RBD-antibodies. Forty-two percent of RBD-antibody positive ME/CFS and 31% of HDs were asymptomatically infected. Furthermore, to evaluate whether the observed higher RBD IgG level in ME/CFS compared with HD, was due to difference in saliva volume, salivation rate or dry mouth, we analyzed total IgG levels in saliva of the two cohorts. Total saliva IgG was found to be higher in HDs compared to ME/CFS (*p=0.0499, **Figure 1E**).

### Differential antibody response against SARS-CoV-2 in saliva and plasma: stratification of the cohorts into systemic and local responders

Since we found a number of study participants presenting with high SARS-CoV-2-specific antibody titers in saliva, but not in plasma, as well as donors who were both saliva and plasma-positive, we stratified each cohort into three groups: 1. Systemic responders (plasma RBD antibodies with or without saliva RBD, designated systemic-ME and systemic-HDs), 2. Local responder (saliva RBD antibodies only, designated local-ME and local-HDs), and 3. Negative RBD-responders (negative-ME and negative-HDs).

**Figure 2:**
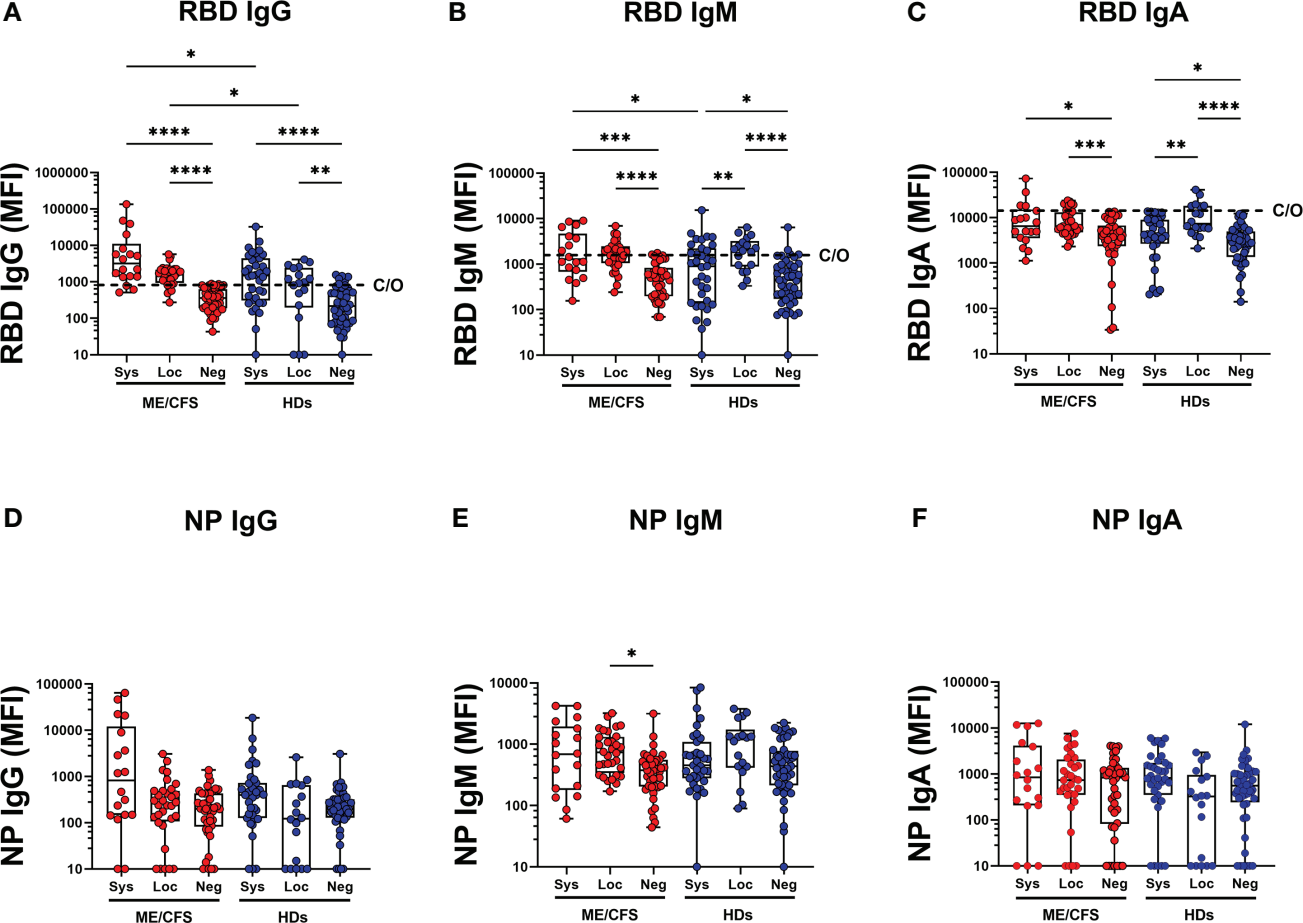
SARS-CoV-2 RBD and NP antibodies in systemic and local responders in ME and HDs. **(A)** SARS-CoV-2-RBD (RBD) for IgG, **(B)** IgM and **(C)** IgA class. SARS-CoV-2 NP (NP) for **(D)** IgG, **(E)** IgM and **(F)** IgA class. Systemic: Participants RBD-positive for systemic response in plasma. Local: Participants RBD-positive for local response in saliva. Negative: Participants patients RBD-negative both in plasma and saliva. Data are presented as boxplots with median values and 25^th^ and 75^th^ percentile. MFI, median fluorescence index. Statistically significant differences according to nonparametric Kruskal/Wallis procedure and false discovery rate adjustment (5%), are indicated as *p < 0.05, **p < 0.01, ***p < 0.001 and ****p < 0.0001. Dashed horizontal lines marked C/O in **(A-C)** indicate saliva cut-off levels as explained in **Figures 1** legend.

SARS-CoV-2 RBD and NP antibodies were analyzed in systemic and local responders in ME and HDs. Saliva RBD-IgG levels were significantly higher in patients with ME/CFS compared with HDs both in local and systemic responders (*p=0.0176 and *p=0.0136, respectively) (**Figure 2A**, **Table S3**, **S7**). Regarding SARS-CoV-2 nucleoprotein (NP IgG, IgM, IgA), no differences were found between groups (**Figures 2D, E, F**). Within the cohort of HDs, a higher saliva RBD IgM/IgA-response was observed in local responders compared with systemic responders: local-HD vs systemic-HD: IgM: **p=0.0062, IgA: **p=0.0069; **Figures 2B, C**, **Table S2B**). This observation underlines the importance of IgA and IgM in the innate mucosal B cell antiviral defense.

### Reactivation of latent herpesviruses EBV and HHV6A, and human endogenous retrovirus HERV-K in the oral mucosa after mild/asymptomatic COVID-19

**Figure 3:**
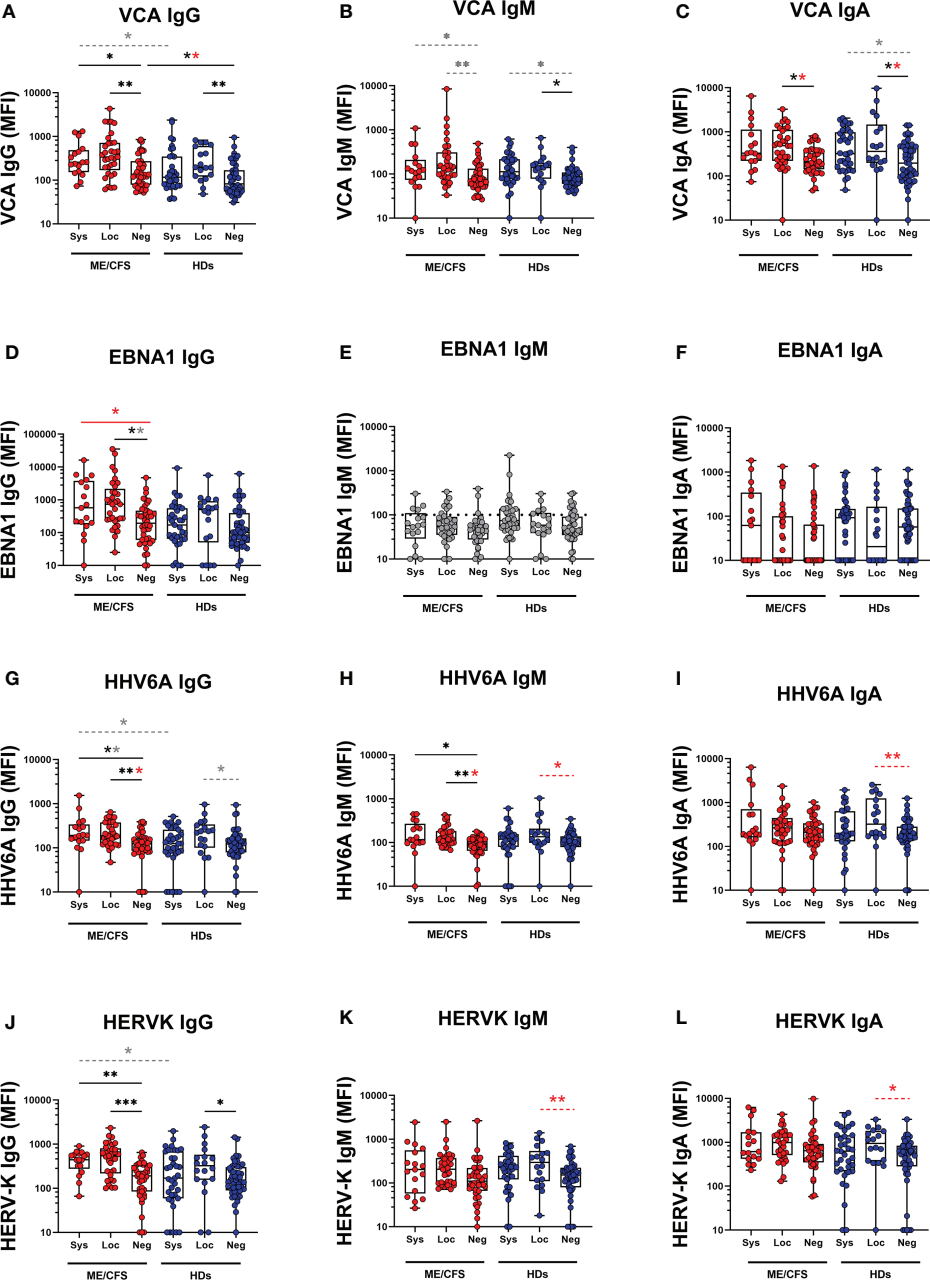
Saliva antibody reactivity to herpesviruses and endogenous retrovirus HERV-K in patients with ME/CFS (ME) and healthy donors (HDs). **(A)** Epstein-Barr virus viral capsid protein (VCA) IgG, **(B)** VCA IgM, **(C)** VCA IgA, **(D)** Epstein-Barr nuclear antigen 1 (EBNA1) IgG, **(E)** EBNA1 IgM, **(F)** EBNA1 IgA, **(G)** human herpes virus 6A (HHV6A) IgG, **(H)** HHV6A IgM, **(I)** HHV6A IgA, **(J)** human endogenous retrovirus K (HERV-K) IgG, **(K)** HERV-K IgM, **(L)** HERV-K IgA. Sys, participants RBD-positive for systemic response in plasma. Loc, participants RBD-positive for local response in saliva. Neg, participants RBD-negative both in plasma and saliva. Data are presented as boxplots with median values with 25^th^ and 75^th^ percentile. MFI, median fluorescence index. Statistically significant differences according to non-parametric Kruskal-Wallis procedure and false discovery rate adjustment (5%), are indicated as *p < 0.05, **p < 0.01, ***p < 0.001; *ns*, non-significant. Dimmed dots and dashed line indicates absence of antibodies (assay background levels). Dimmed/grey *p*-value asteriks* indicate loss of significance after confounding factor (age and gender) analysis with multiple linear regression and adjustment for FDR of 5% according to Benjamini, Krieger, Yekutieli. Red p-values asteriks* indicate gain of significance after adjustment, and black p-value asteriks* indicate no change after adjustment.

**Figure 4:**
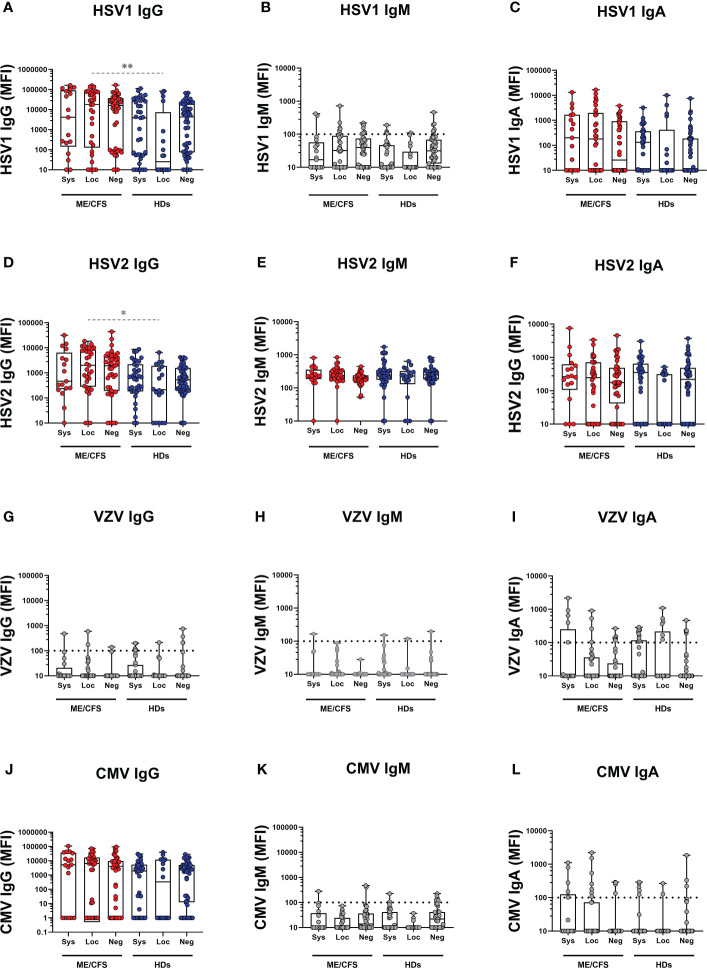
Saliva antibody reactivity to herpesviruses in patients with ME/CFS (ME) and healthy donors (HDs). **(A** herpes simplex-1 virus (HSV1) IgG, **(B)** HSV1 IgM, **(C)** HSV1 IgA, **(D)** herpes simplex-2 virus (HSV2), **(E)** HSV2 IgM, **(F)** HSV2 IgA, **(G)** varicella zoster virus (VZV), **(H)** VZV IgM, **(I)** VZV IgA, **(J)** human cytomegalovirus (CMV) IgG, **(K)** CMV IgM, **(L)** CMV IgA. Systemic: Participants RBD-positive for systemic response in plasma. Sys, participants RBD-positive for systemic response in plasma. Loc, participants RBD-positive for local response in saliva. Neg, participants RBD-negative both in plasma and saliva. Data are presented as boxplots with median values with 25^th^ and 75^th^ percentile. MFI, median fluorescence index. Statistically significant differences according to non-parametric Kruskal-Wallis procedure and false discovery rate adjustment (5%), are indicated as *p < 0.05, **p < 0.01 Dimmed p-value asteriks* indicate influence of confounding factor. Dimmed dots and dashed line indicates absence of antibodies (assay background levels). Dimmed/grey *p*-value asteriks* indicate loss of significance after confounding factor (age and gender) analysis with multiple linear regression and adjustment for FDR of 5% according to Benjamini, Krieger, Yekutieli.

Potential contribution of SARS-CoV-2 infection to latent virus reactivation was evidenced by antibody ‘fingerprint’ analysis in saliva. Specifically, IgG, IgM, and IgA class of anti-viral antibodies against a panel of six human herpesviruses 1-6 (HHV1-6: HSV1, HSV2, VZV, EBV, CMV, HHV6A), and human endogenous retrovirus-K (HERV-K) were investigated. The results demonstrate a distinct pattern of latent virus reactivation in saliva following mild/asymptomatic SARS-CoV-2 infection in patients with ME/CFS compared to HDs (**Figure 3** and **Figure 4**).

The systemic responding ME group showed significant upregulation of IgG levels against EBV viral capsid antigen (VCA) and EBNA1, as well as IgG and IgM against HHV6A compared with the negative-ME group (**Figures 3A, D, G, H, J**; **Table S2A**, **S6**). In contrast, the corresponding systemic-HD group showed no elevation of anti-viral antibodies compared to negative-HDs (**Figure 3**, **Table S2B**, **S5**).

In the locally responding ME group, a significant upregulation of anti-viral antibody levels was noted against EBV (VCA IgG, VCA IgA, EBNA1 IgG), HHV6A (IgG and IgM), and HERV-K (IgG) versus the negative-ME group (**Figures 3A, C, D, G, H, J**; **Table S2A**, **S6**). In the corresponding local-HD group, significant upregulation of anti-viral antibody levels was noted against EBV (VCA IgG, VCA IgM, VCA IgA), HHV6A (IgM, IgA), and HERVK IgG, IgM, IgA (**Figures 3A–C, H–L**; **Table S2B**, **S5**) compared with negative-HD.

### Latent virus reactivation is more pronounced in ME/CFS compared with HDs

The differential effect of SARS-CoV-2 infection on latent virus reactivation *between* the two cohorts of patients with ME/CFS and HDs was investigated by comparing pairs of either systemic or local-responders. We first found that in systemic-ME, VCA IgG, HHV6A IgG, and HERV-K IgG levels, were significantly higher compared to the systemic-HDs (**Figures 3A, G, J**, **Table S3**). Also, in the saliva-responder groups, HSV1 IgG and HSV2 IgG titers were significantly higher in the local-ME than in local-HDs (**Figure 4D**, **Table S3**). However, after adjusting for age and gender as confounding factors, there was no statistical differences detected for HSV1 IgG and HSV2 IgG (**Figure 4**, **Table S7**). Analysis of IgM antibody titers did not show any differences. The two cohorts showed absence of specific antibodies (e.g. assay background levels) for EBNA1 IgM, HSV1 IgM, VZV (IgG, IgM, IgA), CMV (IgM, IgG) (**Figure 3**, **Figure 4**).

### Testing the influence of age and gender difference on antiviral antibody levels

**Figure 5:**
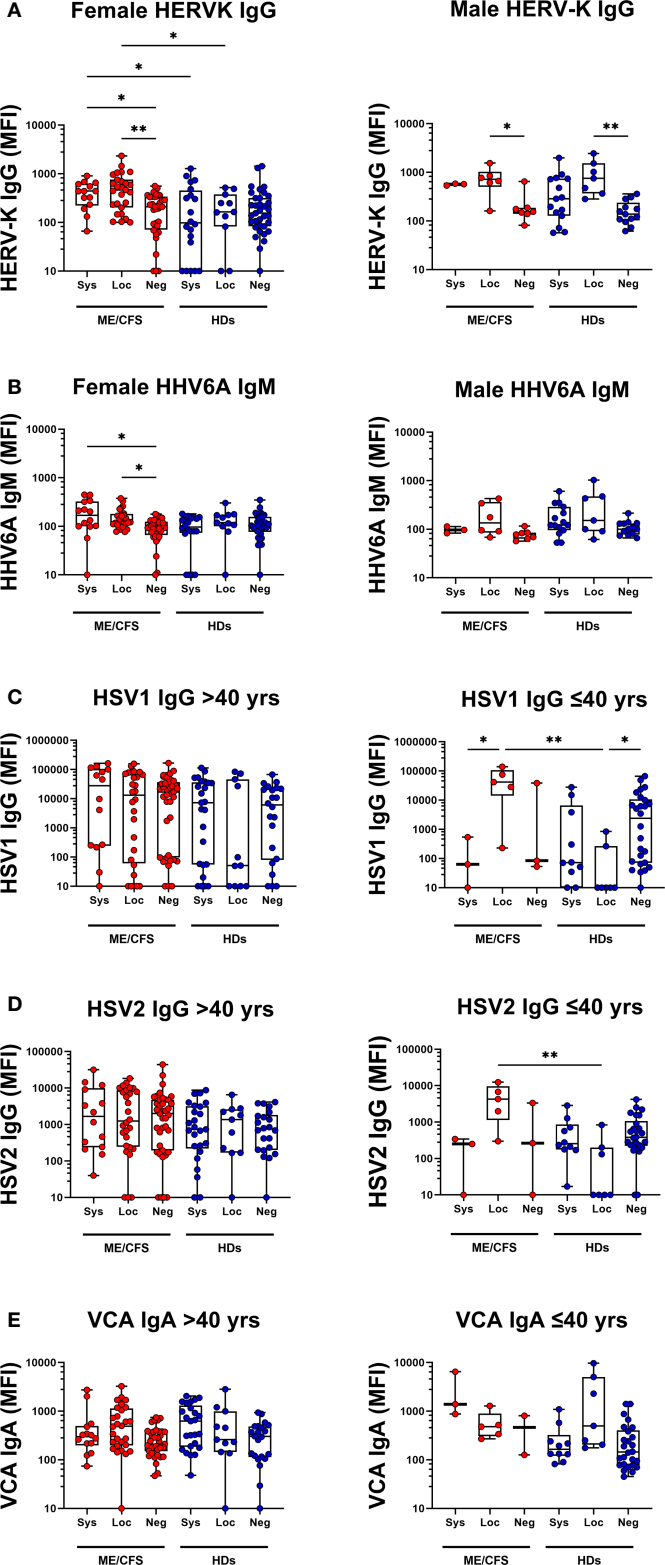
Female vs Male saliva and age-based antibody profile comparisons in patients with ME/CFS (ME) and healthy donors (HDs). Male/Female IgG responses against **(A)** human endogenous retrovirus K (HERVK) and **(B)** IgM responses against human herpesvirus 6A (HHV6A) in all female and male participants. **(C)** Age-dependent IgG responses against herpes simplex virus 1 (HSV1) for participants under 40 years of age (left graph) and over 40 years of age (right graph). IgG responses against herpes simplex virus 2 (HSV2) **(D)** for participants under 40 years of age (left graph) and over 40 years of age (right graph). **(E)** IgA responses against VCA for participants under 40 years of age (left graph) and over 40 years of age (right graph). Sys, participants RBD-positive for systemic response in plasma. Loc, participants RBD-positive for local response in saliva. Neg, participants RBD-negative both in plasma and saliva. Data are presented as boxplots with median values with 25^th^ and 75^th^ percentile. MFI, median fluorescence index. Statistically significant differences according to non-parametric Kruskal-Wallis procedure and false discovery rate adjustment (5%), are indicated as *p < 0.05, **p < 0.01.

The ME/CFS and HD cohorts differed in gender distribution (ME/CFS had 82% females vs. 65% in HDs) and age distribution (ME/CFS mean age 52 ± 11 yrs vs. 44 ± 13 for HDs): Therefore, we performed multiple regression analysis for each dependent (antibody) variable (n=16) using Benjamini, Hochberg, Yekutieli FDR of 5%. **Tables S5**, **S6** and **S7** show data from statistical analysis within and between HD and ME/CFSs cohorts following the correction for age and sex (see also **Figure 3** and **Figure 4**). First, we found Neg-HDs vs Loc-HDs: RBD IgG is higher in males (**p=0.0027), RBD IgM is lower with increasing age (**p=0.0016). Loc-HDs vs Sys-HDs: RBD IgA is higher in males (*p=0.0172). Neg-ME vs Loc-ME: RBD IgA is lower in males (*p=0.0257). Loc-ME vs Sys-ME: RBD IgM Is lower in males (*p=0.0337). Secondly, in analysis of all the participants in the ME/CFS and HD cohorts, 2 of the 16 different measured antibody responses gender was a significant confounding factor, in 14/16 gender was not statistically significant. For HERV-K IgG and HHV6A IgM, the female and male showed different antibody profiles (**Figures 5A, B**). In particular ME/CFS females showed a stronger elevation of HERV-K IgG vs. males. However, due to low sample size, this must be cautiously interpreted. In 2/16 antibodies, age was a significant confounding factor: HSV1 IgG, and HSV2 IgG. We also analyzed age as a confounding factor by inserting age as a continuous variable. Age was found to be a confounding factor. Detailed analysis of age distribution showed that HD had several participants in the age-span 30-40 year of age, whereas ME/CFS cohort did not. For this reason, we stratified the 2 cohorts into age intervals and found that all the age differences related to anti-viral titers were to be found in participant < 40 years of age, but there was no difference in subgroups in participants > 40 years of age (**Figures 5C–E**). We also analyzed whether a previous history of infectious mononucleosis (IM), and/or medication with anti-viral or corticosteroids, would affect the antibody responses against the latent viruses included in this study. The 39 ME/CFS participants with a history of IM as a disease trigger (disease mean duration 13.0 years) were compared with patients without a history. No significant difference was found. Fourteen percent (14%) of ME/CFS participants reported medication by anti-viral drugs such as aciklovir and/or corticosteroids vs. 3% in the HD cohort. Statistical testing of antiviral titers in persons with vs. without these drugs did not show any differences (p>0.05).

**Figure 6:**
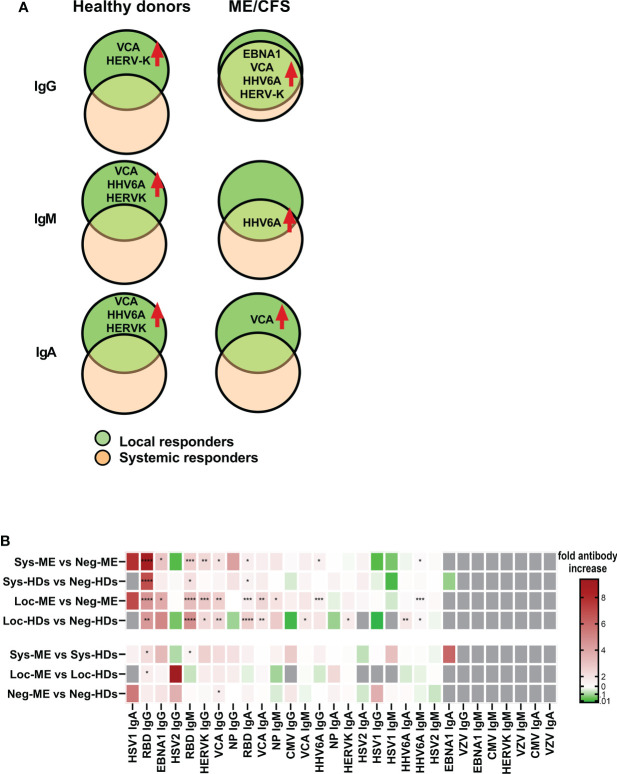
Summary of the effect of SARS-CoV-2 infection, regarding latent virus reactivation. **(A)** Comparison within each cohort based on IgG, IgM, and IgA antibody response fingerprint in the oral mucosa. Only antibody responses with statistically significant differences are displayed on charts. Local responders: Participants RBD-positive for local response in saliva. **(B)** Hierarchical clustered heatmap showing fold-change of saliva antibody titers in local and systemic responses within and between each group including *p*-values. Statistically signicant differences according to nonparametric Kruskal/Wallis procedure and false discovery rate adjustment (5%), are indicated as *p < 0.05,**p < 0.01, ***p < 0.001 and ****p < 0.0001.

Differences in saliva antibody titers (fold-change) in local and systemic responses relative to the corresponding negative groups, highlights the augmented responses to EBV, HERV-K and HHV6 are shown in **Tables S5**, **S6**, **S7**. A summary of the effect of SARS-CoV-2 infection, regarding latent virus reactivation *within* and *between* each cohort (based on IgG, IgM, and IgA antibody response fingerprint in the oral mucosa) show differences within respective cohort but not between cohorts (**Figures 6A, B**). Hierarchical clustered heatmap showing fold-change of saliva antibody titers in local and systemic responses relative to the corresponding negative groups, highlights the augmented responses to EBV, HERV-K and HHV6 (**Figure 6B**, **Tables S5**, **S6**, **S7**).

### Local reactivation of latent viruses was confirmed in pairwise analysis by following individuals before and after SARS-CoV-2 infection

**Figure 7:**
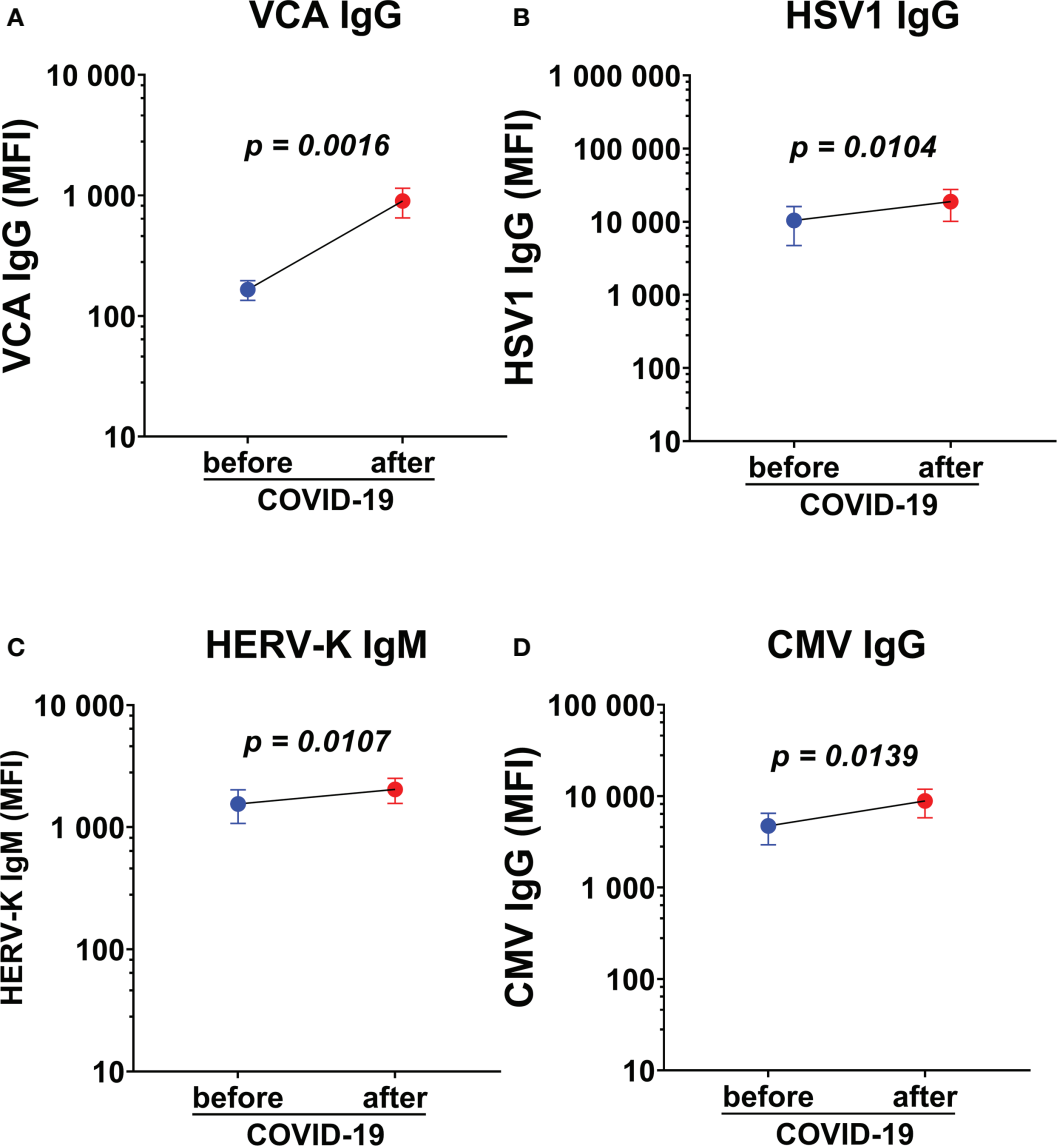
Paired analysis of saliva antibody reactivity to herpesviruses before and after SARS-CoV-2 infection. **(A)** Epstein-Barr viral capsid antigen (VCA) IgG, **(B)** herpes simplex-1 virus (HSV1) IgG, **(C)** endogenous retrovirus (HERV-K) IgM, and **(D)** cytomegalovirus (CMV) IgG in the same individuals before and after SARS-CoV-2 infection (n=19). Data are presented as mean antibody values with SEM of 19 individuals before and after infection. MFI, median fluorescence index. Statistically significant differences according to paired Wilcoxon-signed rank test are indicated in the graphs.

Paired analysis of saliva antibody reactivity to herpesviruses before and after SARS-CoV-2 infection were analyzed in a subgroup of participants (n=19, 15 HDs and 4 ME/CFS), who were infected with SARS-CoV-2 after the first round of sampling and during the course of this study (in the second pandemic wave between December 2020 to January 2021). Infection was documented by either PCR and/or established specific symptoms and was confirmed by the significant upregulation in RBD IgG (data not shown*, p=0.03*) and IgM response in paired samples (data not shown*, p=0.04*). We found significant upregulation of VCA IgG, HSV1 IgG, HERV-K IgM, CMV IgG, within the same individuals when comparing antibody levels before and after SARS-CoV-2 infection (**Figures 7A–D**). The limited sample size does not allow any conclusion on whether the increase is mote in ME/CFS cf. HD.

### Baseline antibody responses against latent viruses in the local oral mucosa is augmented in patients with ME/CFS

To evaluate the status of latent viral reactivation independently of SARS-CoV-2 infection, we compared the negative-ME to the negative-HD. In negative-ME, only VCA IgG was significantly higher compared with the negative-HD (**Figure 3A**, **Table S7**).

### SARS-CoV-2 infection generates a distinct antibody fingerprint of latent virus reactivation in saliva, but not in plasma

**Figure 8:**
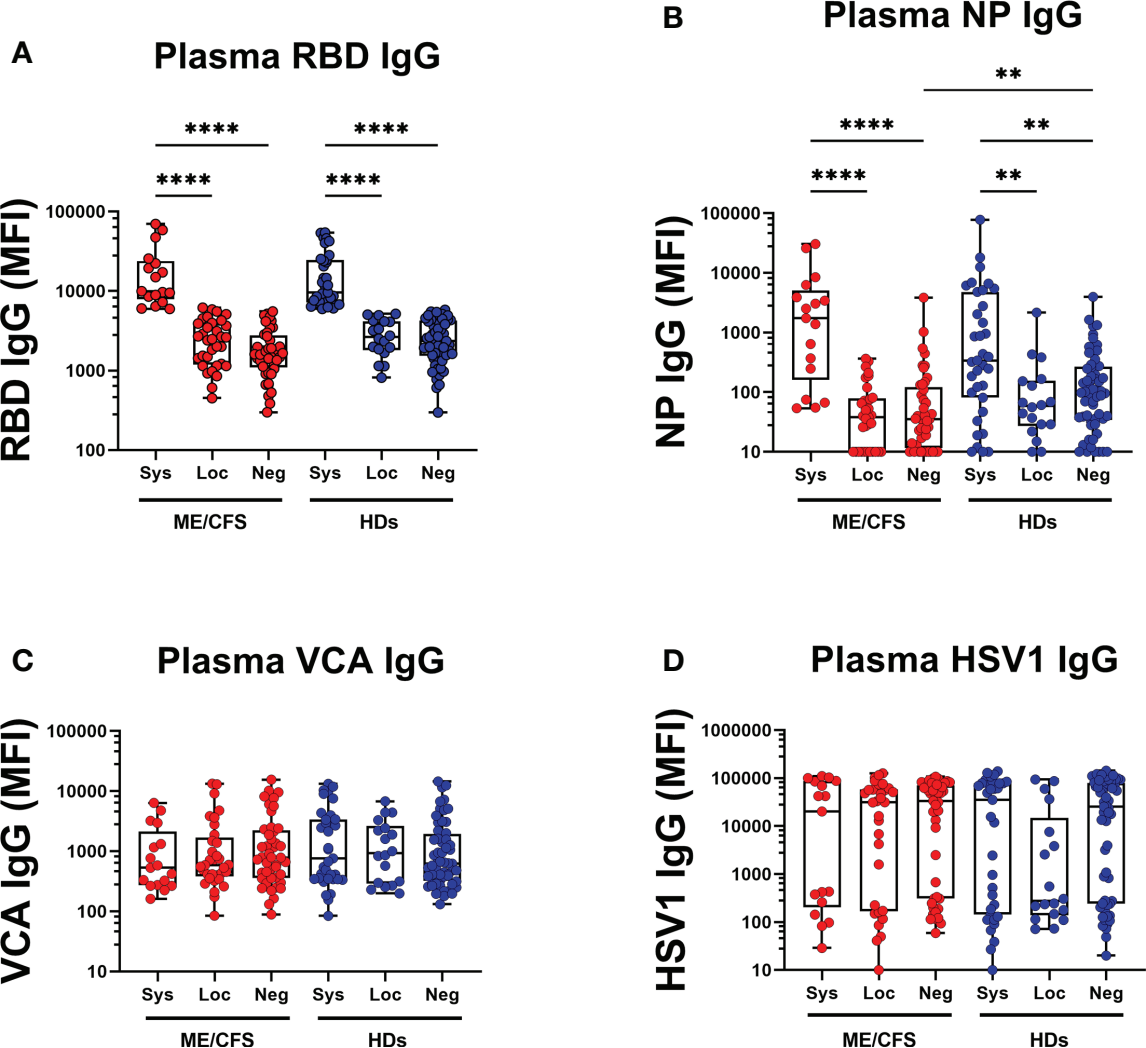
SARS-CoV-2 infection generates a distinct antibody fingerprint of latent virus reactivation in saliva but not in plasma. **(A)** SARS-CoV-2-RBD (RBD), **(B)** SARS-CoV-2 NP (NP), **(C)** Epstein-Barr viral capsid antigen (VCA), and **(D)** herpes simplex virus-1 (HSV1) in patients with ME/CFS (ME) and healthy donors (HDs). Sys, participants RBD-positive for systemic response in plasma. Loc, participants RBD-positive for local response in saliva. Neg, participants RBD-negative both in plasma and saliva. Data are presented as boxplots with median values with 25^th^ and 75^th^ percentile. MFI, median fluorescence index. Statistically significant differences according to non-parametric Kruskal-Wallis procedure and false discovery rate adjustment (5%), are indicated as **p < 0.01, and ****p < 0.0001.

Finally, we determined whether antibody responses against SARS-CoV-2 and latent viruses were equivalent in the local oral mucosa (saliva) and systemically in plasma. RBD IgG, NP IgG, VCA IgG and HSV1 IgG were analyzed in the two compartments. Regarding SARS-CoV-2 responses, RBD IgG and NP IgG in plasma (**Figures 8A, B**, **Table S4A, B**) correlated with RBD IgG response in saliva (**Figures 2A, D**, **Tables S2A, B**, **S3**, **S5**, **S6**, **S7**) of systemic responders. On the contrary, VCA IgG and HSV1 IgG did not show any significant difference in plasma (**Figures 8C, D**), whereas saliva generated a distinct antibody fingerprint consistent with latent EBV reactivation as seen by elevated VCA-antibodies. Median HSV-1 IgG levels were elevated but did not reach significance. (**Figure 3A**, **4A**, **Table S5, S5, S7**).

## Discussion

We provide evidence that mild/asymptomatic SARS-CoV-2 infection triggers reactivation of latent symbiotic viruses as detected by antibody responses locally in the oral mucosa. This response was not observed systemically in plasma. The anti-viral antibody signature is distinct between patients with ME/CFS and HDs. Firstly, anti-EBNA1 elevation is unique for ME/CFS. Secondly, in ME/CFS reactivation of latent viruses is present both in local and systemic responders. There is an overlapping antibody signature observed: compared to HDs, the patients with ME/CFS had elevated antibodies at baseline for VCA IgG, e.g. without SARS-CoV-2 infection.

The frequency of asymptomatic infection has recently been estimated to be higher than anticipated. In our study, 42% of the patients with ME/CFS and 31% of HDs were found to be asymptomatically infected. Up till December 2020, 40.5% among the global population with confirmed COVID-19 were asymptomatic (29). Since we found several study participants that had SARS-CoV-2-specific antibodies in saliva, but not in blood, we stratified our cohorts into local and systemic responders. In the cohort of patients with ME/CFS 35% were local responders and 19% were systemic responders. Corresponding percentages for HDs were 16% and 32%. Remarkably, none of the local HD responders reported symptoms, indicating an effective first-line of innate defense mechanism. For patients with ME/CFS, due to the presence of frequent flu-like symptoms, we could not draw any conclusions regarding COVID-19-related symptoms and relied on blood and saliva antibody levels for the definition of an asymptomatic/unknown infection. It is increasingly realized that local mucosal-innate immunity presents with a distinct signature, including interferon activity, and has important roles in SARS-CoV-2 defense (30, 31).

A more pronounced local mucosal antibody-specific response against SARS-CoV-2 was observed in patients with ME/CFS compared to HDs even though, total IgG levels in saliva were similar. This is consistent with the hypothesis of a hyper-inflammatory response to pathogen-associated molecular patterns (PAMPs), including SARS-CoV-2 spike protein-1, in patients with multiple chronic diseases (32). In terms of cell-mediated immune responses though, patients with ME/CFS exhibit perturbations that include unresponsive natural killer cells (33), decreased CD8^+^ T-cell cytotoxicity (34) and activation, as well as increased (24)percentage of regulatory T cells (35). While patients with ME/CFS have enhanced local responses against SARS-CoV-2, systemic responses in plasma were similar to those of HDs. In a recent study, samples from patients with ME/CFS have demonstrated altered methylation and gene expression levels for the ACE and *ACE2* locus, suggesting that the patients may have a higher risk of being infected with SARS-CoV-2 (36).

Noteworthy, gender had a significant influence on 2/16 antiviral responses, but in 14 of the 16 no statistical significance was found. HERV-K IgG and HHV6A antibody responses in female ME/CFS participants showed a more pronounced elevation vs. males. This observation renders further studies, and due to low sample size, the gender data should be cautiously interpreted. The influence of age on the saliva anti-viral responses was significant for 2/16 antibodies: HSV1 IgG and HSV2 IgG as detected by multiple linear regression analysis and subgrouping the cohorts in <40 years and >40 years of age (**Figures 6**). However, a previous medical history of infectious mononucleosis, and/or medication with anti-viral drugs or corticosteroids, did not have any effect on antibody responses against reactivated latent viruses. The influence of age on local mucosal anti-viral responses has been observed by others (24), but renders further detailed studies.

Reactivation of latent viruses can be triggered by numerous factors including exogenous viral infections, trauma, environmental factors, and mental stress, and as part of the aging process. Severe SARS-CoV-2 infection was recently associated with herpesvirus reactivation (HSV1, VZV, EBV, CMV, and HHV6), in hospitalized or ICU-treated patients (19). In our study, all infected participants presented with a mild/asymptomatic form of the infection. Still, COVID-19 triggered reactivation of EBV, HHV6 and HERV-K both in ME/CFS and HDs (**Figure 6**). Significant upregulation of antibodies against EBV and HERV-K was also observed in individuals who were analyzed before and after COVID-19 (**Figure 7**).

EBV infects almost all humans during their lifetime and, following the acute phase, the virus persists lifelong. EBV infects B cells leading to a latent residence in non-dividing resting memory B cells as an episome. Upon reactivation, viral particles are released into oral mucosa. These are known to cause polyclonal activation of B-cells followed by immunoglobulin secretion (37). Long-term B-cell activation may constitute an increased risk for triggering autoimmune responses (38) (39). High rate of active EBV infection has been observed among patients with ME/CFS suggesting, at least in a subset of cases, that EBV is an important factor for the development of the disease (23). Whether EBV is a mere ‘initiator’ of ME/CFS or also a ‘driver’ of the disease, remains to be clarified. This hypothesis though, is reminiscent of EBV involvement in multiple sclerosis where EBNA1 was recently identified as the ‘driver’ (40). Remarkably, also in the current study, EBNA1 stands out as a unique entity in the ME/CFS cohort, causing significant local anti-EBNA1 IgG release after COVID-19. Since anti-EBNA1 elevation was not found in HDs, it is of significant importance to follow-up this finding. A high VCA titer indicates current or past exposure, and a high EBNA1 IgG may indicate a long-term release of EBNA1-DNA complexes from apoptotic EBV+ B cells (41). Several questions remain to be answered: Does anti-EBNA1 generate cross-reacting autoantibodies (antigenic mimicry), similar to the situation in multiple sclerosis? (40), What is the mechanism behind the fact that despite the augmented anti-viral responses observed in patients with ME/CFS, their immune defense is unable to strictly surveil and control the reactivation of EBV?

A previous study from our group evaluated IgG antibody responses systemically against herpesviruses in patients with ME/CFS versus healthy donors (25). Although no significant differences were noted, minor relative differences between antibody reactivities indicated that the immune system of some patients interact with the ubiquitous herpesviruses in a way different from that of healthy controls (25). An elevated production of EBV and HHV6A dUTPase was recently demonstrated in patients with ME/CFS and suggested to induce T follicular helper cell differentiation, which is critical for high-affinity antibodies and long-lived plasma cells (42). In this study, we provide evidence for significantly stronger saliva antibody responses against latent viruses in both systemic-ME and local-ME after COVID-19. Importantly, non-infected patients with ME/CFS, had significantly elevated IgG responses against latent EBV VCA compared to non-infected HDs, signifying a higher ‘baseline’ status of viral reactivation. Elevated antibody responses against herpesviruses in patients with ME/CFS deserve further attention. A plausible scenario is that patients with ME/CFS, and possibly PASC, have immune cells that reside in a state reminiscent of senescence. Senescent cells have been suggested to alter responses to PAMPs and contribute to a heightened but aberrant immune response. Those immune responses involve increased production of pro-inflammatory cytokines and chemokines by innate immune cells that further amplify the senescent phenotype (32). Sato et al. (43) recently found a biased B-cell repertoire in patients with ME/CFS reporting an infectious/viral trigger of the disease. This finding also correlated with an upregulation of interferon (IFN) inducible genes (IFN signature) in antibody producing plasma-blasts, which is a hallmark of viral infections.

In the present study, mild/asymptomatic SARS-CoV-2 infection was found to upregulate antibody responses against proteins of HERV origin both locally and systemically. This is supported by parallel studies showing that exogenous viral infections including SARS-CoV-2, can trigger transcription of HERVs and suggested to aid in the defense against newly invading pathogens (44). Conversely, prior distinctive HERV expression patterns could modulate exogenous SARS-CoV-2 infection and have been proposed to account for differential SARS-CoV-2 severity and symptoms (45). In our study, immune responses against HERV-K were more prominent in patients with ME/CFS. Significantly heightened IgG responses against HERV-K were found uniformly in all three subgroups of patients with ME/CFS versus the respective HDs. This is in line with previous reports on upregulated HERV-K gene expression in PBMCs (46) of patients with ME/CFS, and epigenetic studies demonstrating extensive hypomethylation of non-coding genetic elements (47). The physiological significance of elevated antibody responses against HERV-K remains to be determined. HERVs are integrated in the germline and inherited in Mendelian fashion and IgM antibody responses against HERV antigens have been proposed to represent natural antibodies (48). Ancestral retroviral envelope proteins have been suggested to regulate herpesvirus reactivation and persistence in the latent state (49), providing a possible link to the altered herpesviruses’ signature in patients with ME/CFS. Recently, a cumulative role for EBV, HERV-K/W, and HHV-6 was proposed in driving the inflammatory cascade in multiple sclerosis (41). Further studies are needed to analyze whether SARS vaccine will reactivate latent viruses similar to what is observed in transplant patients (50).

One of the main findings of the present study is that the distinct antibody fingerprint of latent virus reactivation found in saliva was not detected in plasma. This is evident by the lack of significant difference in group comparisons, contrary to the strong statistical differences in saliva (**Figures 3**, **4**, **8**). Herpesviruses are commonly found in the oral cavity (51), therefore their reactivation and subsequent immune responses are easily traceable in saliva, as demonstrated in our study. The triggering event responsible for viral reactivation may not be robust enough in the case of mild/asymptomatic versus severe SARS-CoV-2 infection. Therefore, the antibody signature following mild/asymptomatic infection may be confined locally and hence not detectable in plasma. In contrast to latent virus responses, the response to SARS-CoV-2 in saliva samples correlate with those in plasma. Our results further support the use of saliva samples when investigating anti-SARS-CoV-2 antibodies (52–54). Furthermore, we propose that saliva samples are preferable when analyzing antibody responses against latent viruses.

Our findings demonstrate that SARS-CoV-2 infection even in its mild/asymptomatic form is a potent trigger for reactivation of latent herpesviruses and endogenous retroviruses. This is particularly relevant for individuals suffering from ME/CFS, since they have elevated immune responses against latent viruses. Furthermore, SARS-CoV-2 infection in ME/CFS imposes both a unique and an augmented antibody fingerprint, adding further evidence for altered immune responses in the syndrome. These alterations may compromise the host defense when encountering primary/exogenous viral infections including COVID-19. If the same phenomenon could also be demonstrated in PASC, it could be a candidate mechanism accounting for the prolongation of symptoms. The findings can have important clinical implications as well. Our results can contribute to setting immunological tests that are easy to collect and may strengthen the diagnosis of ME/CFS and possibly PASC. Furthermore, our results highlight that treatment options directed to boost antiviral immune responses, may benefit patients with ME/CFS by tuning the fine balance between latent virus reactivation and an appropriate immune response.

## Materials and methods

*Study Design*. Study participants were non-vaccinated and enrolled during the second half of 2020. Healthy donors were enrolled by announcements at Linköping University and Hospital. The ME/CFS cohort was enrolled amongst patients at the Brageé Clinic diagnosed with ME/CFS, using the Swedish national digital health care 1177.se guide for surveys. All patients were diagnosed before the COVID-19 pandemic. Follow-up samplings and interviews and questionnaire-responses were conducted at 3-month intervals for both cohorts. Data from the follow-ups were used in this study for the determination of individuals that seroconverted during that period from negative to SARS-CoV-2 positive. These pre-infection saliva samples served as a cut-off baseline criterion for the SARS-CoV-2 antibody positivity.

*Study population.* Cohort 1 consisted of 95 patients with ME/CFS and included post-COVID patients recovered from mild SARS-CoV-2 infection and non-exposed patients. Patients with ME/CFS included in this study were diagnosed according to the 2003 Canadian Consensus Criteria (1) at the Bragée Clinic, Stockholm with exclusion of other medical or neurological diseases. Cohort 2 consisted of 110 healthy donors with no prior diagnosis for ME/CFS (cohort designation: HDs) and included participants having had mild to moderate COVID-19 symptoms, as well as non-exposed individuals. Pre-COVID-19 plasma samples (n=50) were collected in 2015 from anonymous healthy blood donors at Linköping University Hospital (termed BD2015) and were used to define the cut-off levels for SARS-CoV-2 negative and positive samples.

The exclusion criteria for participant enrollment were existence of current active infection and/or infectious disease symptoms and age below 18 years. Thus, participants had no evidence of active SARS-CoV-2 or other infection at the time of sampling. All study participants actively approached us and were enrolled in a consecutive order. Recovered COVID-19 participants had presented with either mild or asymptomatic infection at the time of the disease, and none had been admitted to the hospital. Disease severity in patients with ME/CFS was assessed by a physician in a 1 (mild) to 4 (very severe) scale. Information related to ME/CFS trigger events (infection, trauma, stress, vaccination or other), disease duration and past infections were retrieved *via* self-reported questionnaire. Demographics and clinical characteristics are summarized in **Table 1**. Study participants were enrolled consecutively (randomly) with no bias/no selection. Female/male gender ratio (4/5) and age distribution (51 ± 11 yrs) agrees with epidemiological studies on ME/CFS in Europe describing that at least 2/3 of the cases are women in their most productive phases of life (55, 56).

**Table 1:** Demographics and clinical characteristics.

	ME/CFS individuals (n=95)	Healthy individuals (n=110)
**Female % (n/total)**	82% (78/95)	65% (71/110)
**Age** (yrs), mean ± SD (range)	51 ± 11 (21-75)	44 ± 13 (18-79)
**Past Infections**		
Infectious mononucleosis n (%)	39 (41%)	7 (6%)
**Disease Duration** (years) mean (range)	13.0 (1.0-44)	n/a
**Disease Severity** (1-4 Scale) 1=mild 2=moderate 3=severe 4=very severe Unknown:	(n=95) 32 50 10 0 3	n/a
**ME/CFS trigger event**		
Infection n (%)	55 (58%)	n/a
Trauma n (%)	16 (17%)	n/a
Stress n (%)	26 (27%)	n/a
Other n (%)	10 (11%)	n/a
Unknown n (%)	12 (16%)	n/a

n/a, not applicable.

*Blood samples.* Peripheral blood was collected in 10-mL EDTA tubes (Cat#10331254, BD Vacutainer, Fisher Scientific, Göteborg, Sweden). Up to 10 mL of whole blood was used for plasma separation by centrifugation (2,000 *g*, 15 min, 4°C) and aliquots were stored at -80°C until further analysis. Blood samples were collected at the same visit as saliva samples.

*Saliva samples.* Prior to saliva collection, participants were asked to rinse their mouth with water and confirm that they had fasted, refrained from smoking, or chewed a gum during the previous hour. They were asked to document oral disease or injury. They should not have taken oral medication, not brushed the teeth for a minimum of 1 h before sampling and no dental work was performed within 24 h prior to sample collection. Donors were asked to provide a 5 mL saliva sample into a 50 mL sterile conical tube by passive drool. Follow-up samplings were conducted every 3 months during a year, then saliva samples were collected using Saliva Bio Swabs (Salimetrics, Carlsbad, CA) according to the manufacturer instructions. Briefly, the participants were instructed to position the cotton swab in the mouth for 4 min. The saturated swab was then transferred into a 15 mL storage tube, and either was frozen immediately or stored/transported on ice upon receipt of the laboratory for processing. Samples were centrifuged (2600*g*, 30 min, 4°C) to separate cells and insoluble matter. The supernatant was removed and complemented with 1/1000 v/v complete^™^ protease (Cat#11836170001, Sigma-Aldrich Sweden AB, Stockholm, Sweden) and Pierce™ phosphatase inhibitor cocktails (Cat#88667, Thermo Scientific, Göteborg, Sweden), subsequently aliquoted and stored at -80°C till analysis. On the day of the assay, samples were thawed and micro-centrifuged (2600 g, 30 min, 4°C) prior to analysis.

*Antibody analysis in blood and saliva.* Suspension multiplex immunoassay (SMIA) analysis was performed using MagPlex^®^ microspheres (Luminex Corp., Austin, TX) for the coupling of antigens according to the manufacturer’s protocol. Briefly, 200 µL of the stock microsphere solution (1.25 × 10^7^ beads/mL) were coupled by adding either 10 μg of recombinant protein antigen (**Table S1**) diluted in phosphate buffered saline (PBS: 0.15 M sodium chloride, 10 mM sodium phosphate, pH 7.4) containing 0.05% (v/v) Tween 20 and incubated for 15 min on a rocking shaker at room temperature (RT). The beads were then washed with 0.5 mL StabilGuard solution (Cat#SG01-1000, SurModics, Eden Prairie, MN) using a magnetic separator (Cat#40-285, Milliplex^®^ MAG handheld magnetic separation block for 96-well plates, Millipore Corp. MO) and resuspended in 400 µL of StabilGuard solution. The coupled beads were stored at 4°C in the dark till further use. A complete list of the coupled recombinant protein antigens, antibodies and secondary antibodies is given in **Table S1**.

For blood samples, 50 µL of plasma diluted 1/1000, and for saliva samples 50 µL of sample diluted 1:2.5 in PBS-T containing and 1% (v/v) BSA (Cat# SRE0036, Sigma-Aldrich Sweden AB, Stockholm, Sweden) (PBS-T+1% BSA) was added per well of a flat bottom, 96-well µClear non-binding microtiter plate (Cat#655906, Greiner Bio-One GmbH, Frickenhausen, Germany). Fifty microliters of a vortexed and sonicated antigen-coupled bead mixture (50 beads/µL suspended in PBS-T) was then added to each well. The plate was incubated in the dark on plate shaker at 800 rpm for 1 h at RT. The wells were then washed twice with 100 µL of PBS using a magnetic plate separator (Cat#40-285, Milliplex^®^ MAG handheld magnetic separation block for 96-well plates, Millipore Corp. MO). The beads were resuspended in 100 µL of 1 µg/mL of either goat anti-human IgG-PE or goat anti-human IgM-PE labelled antibody (**Table S1**) in PBST+1% BSA and incubated for 30 min at RT in the dark with rotation at 800 rpm. The beads were subsequently washed twice with PBS+1% BSA, resuspended in 100 µL of PBS+1% BSA and analyzed in a FlexMap 3D^®^ instrument (Luminex Corporation, Austin, TX) according to the manufacturer’s instructions. A minimum of 100 events for each bead number was set to read and the median value was obtained for the analysis of the data. A naked, non-antigen-coupled bead was included as a blank and a PBS-T+1% BSA well as a negative control.

*Analysis of total IgG in saliva.* Total saliva IgG levels were evaluated using an in-house developed SMIA. Goat anti-Human IgG-Fc affinity purified unconjugated antibodies were coupled to MagPlex^®^ microspheres. SMIA was performed as described above using 2.5 µl of saliva diluted in PBS-T containing 1% (v/v) BSA and goat anti-human IgG-PE for the detection step. Total IgG levels (ng/mL) in saliva were calculated against an optimized standard curve of known concentrations (ng/mL) of human gamma-globulin.

*Statistics.* Data were analyzed for the determination of statistical significance of the observed differences between groups, with a *p* value <0.05 considered as significant. All statistical analyses were performed using the SAS Institute JMP program (v 13.2.1) or GraphPad Prism software (v.9.1.2). For the comparisons between ME/CFS and HDs groups, we used the non-parametric Kruskal-Wallis test for multiple comparisons, and controlled for false discovery rate (5%) by using two-stage Benjamini, Krieger and Yekutieli (BKY) procedure (57). Multiple linear regression was performed for the determination of confounding factors (age, sex, mononucleosis) and controlled for false discovery rate of 5% according to BKY. For comparison before and after infection, paired analysis and Wilcoxon signed rank test was used in a validation group. Statistically significant differences are indicated in the figures as *p < 0.05, **p < 0.01, ***p < 0.001 and ****p < 0.0001.

*Study approval.* All participant enrollment procedures and blood/saliva sampling were performed in accordance to established ethical standards and following a study protocol submitted to and approved by the Regional Ethics Committee (Dnr. 2019-0618). Demographic characteristics, medical data and samples were collected after the study participant had acknowledged that they had understood the study protocol and then provided an informed consent.

## Data availability statement

The raw data supporting the conclusions of this article will be made available by the authors, without undue reservation.

## Ethics statement

The studies involving human participants were reviewed and approved by the Swedish Ethical Review Authority, Regional Ethics Committee (Dnr. 2019-0618). The patients/participants provided their written informed consent to participate in this study.

## Author contributions

EA designed research studies, performed samplings, designed and conducted experiments, analyzed data, and wrote the manuscript. MR designed and conducted experiments, analyzed data. PM collected samples and analyzed data. PS, BCB, OP, BB participated in research study design, were responsible for patient contacts, provided samples and clinical data. AR conceptualized, supervised, funded, and designed the project, analyzed the data, and wrote the manuscript. All authors contributed to the article and approved the submitted version.

## Funding

This study was supported by Swedish Research Council (4.3-2019-00201 GD-2020-138), Swedish Cancer Society (no. 211832Pj01H2/Infection-Autoimmunity-B-lymphoma grant to AR) and local Linköping University funds (AR).

## Acknowledgments

We would like to thank Ms. Ingela Jacobsson for helping with the collection of samples in Linköping University, Dr. Kent Nilsson (MD) for patient interviews, and Ms. Susanne Faxell and Ms. Susanne Belboul for the collection of samples from patients with ME/CFS in the Bragée Clinic in Stockholm. We would like to thank the personnel in flow cytometry core facility in Linköping university, Dr Mats Fredrikson for help with statistical analysis and Mr. Björn Gylemo for heat map construction. Finally, we would like to thank all study participants.

## Conflict of interest

PS, BCB, BB, and OP declare disclosure of interest as having income from Bragée Clinics, and BB being a partial owner.

The remaining authors declare that the research was conducted in the absence of any commercial or financial relationships that could be construed as a potential conflict of interest.

## Publisher's note

All claims expressed in this article are solely those of the authors and do not necessarily represent those of their affiliated organizations, or those of the publisher, the editors and the reviewers. Any product that may be evaluated in this article, or claim that may be made by its manufacturer, is not guaranteed or endorsed by the publisher.
