# Supplementary material for: Saliva antibody-fingerprint of reactivated latent viruses after mild/asymptomatic COVID-19 is unique in patients with myalgic-encephalomyelitis/chronic fatigue syndrome
Source: Front Immunol. 2022 Oct 20;13:949787. doi: 10.3389/fimmu.2022.949787 (PMC9630598; doi:10.3389/fimmu.2022.949787)
Supplement: Supplementary file 1 [file DataSheet_1.docx]

**Supplementary material**

**Table S1.** **Recombinant antigens and secondary antibodies and reagents for total IgG used in SMIA**

| **Antigens** | **Cat. #** | **Company** |
| --- | --- | --- |
| SARS-Co-V-2 Spike protein-RBD | 40592-V08B | SinoBiological Inc., Wayne, PA |
| SARS-CoV-2 Nucleoprotein | 158-40588-V08B-100 | SinoBiological Inc., Wayne, PA |
| EBV VCA | ebv-273 | Prospec Protein specialists, Ness-Ziona, Israel |
| EBV EBNA1 | Ebv-271-a | Prospec Protein specialists, Ness-Ziona, Israel |
| HERV-K | MBS1391552-A | MyBiosource Inc., San Diego, CA |
| HSV1 purified viral lysate strain MacIntyre | 10-145-000 | Advance Biotechnologies Inc., Eldersburg, MD |
| HSV2 Strain G, purified viral lysate | 10-146-000 | Advance Biotechnologies Inc., Eldersburg, MD |
| HHV6A (gB) | CSB-EP338963HJZ | Cusabio Technol LLC, Houston, TX |
| VZV (gE) | MBS485127 | MyBiosource Inc., San Diego, CA |
| CMV (gB) | 10202-V08H1 | SinoBiological Inc., Wayne, PA |
| **Secondary Antibodies** |  |  |
| goat anti-human IgG-PE | 2040-09 | Southern BioTech, Birmingham, AL |
| goat anti-human IgM-PE | 2020-09 | Southern BioTech, Birmingham, AL |
| goat anti-human IgA-PE | 2050-09 | Southern BioTech, Birmingham, AL |
| **Reagents for total IgG analysis** |  |  |
| Human IgG-Fc affinity purified unconjugated antibodies | AS10786 | Agrisera AB, Vännäs, Sweden |
| human Gamma globulin | 027722 | Kabi Pharmacia AB, Stockholm, Sweden |

RBD: Receptor Binding Domain, EBV-VCA: Epstein-Barr virus viral capsid antigen, EBNA1: EBV nuclear antigen-1, HERV-K env (rec): human endogenous retrovirus-K envelope recombinant protein, HSV: herpes simplex virus, HHV6A: human herpes virus 6A, VZV: varicella zoster virus, CMV: human cytomegalovirus, PE: phycoerythrin

**Table S2A. Statistical significance and fold-change in saliva within the ME/CFS cohort (before adjusting for age and gender)**

|  |  | **Systemic-ME vs Local-ME** |  | **Local-ME vs Negative-ME** | | **Systemic-ME vs Negative-ME** | |
| --- | --- | --- | --- | --- | --- | --- | --- |
|  |  | ***p-value*** | **fold-change** | ***p-value*** | **fold-change** | ***p-value*** | **fold-change** |
| **SARS-CoV-2-RBD** | IgG | ns | 2.00 | *<0.0001* | 4.48 | *<0.0001* | 8.97 |
|  | IgM | ns | 0.81 | *<0.0001* | 3.15 | *0.0003* | 2.55 |
|  | IgA | ns | 1.00 | *0.0008* | 1.60 | *0.0287* | 1.60 |
| **SARS-CoV-2-NP** | IgG | ns | 4.09 | ns | 1.00 | ns | 4.07 |
|  | IgM | ns | 0.93 | *0.0120* | 1.97 | ns | 1.84 |
|  | IgA | ns | 1.14 | ns | 0.89 | ns | 1.02 |
| **HERV-K** | IgG | ns | 0.81 | *0.0001* | *2.84* | *0.0036* | 2.30 |
|  | IgM | ns | 0.98 | ns | 1.88 | ns | 1.84 |
|  | IgA | ns | 0.62 | ns | 1.53 | ns | 0.95 |
| **EBV-VCA** | IgG | ns | 0.75 | 0.0040 | 2.57 | 0.0392 | 1.93 |
|  | IgM | *ns* | 0.87 | *0.0013* | 1.85 | *0.0437* | 1.62 |
|  | IgA | ns | 0.68 | *0.0251* | 2.36 | ns | 1.60 |
| **EBNA1** | IgG | ns | 0.77 | *0.0030* | 3.79 | ns | 2.93 |
|  | IgM | na | na | na | na | na | na |
|  | IgA | ns | 4.44 | ns | na | ns | na |
| **HHV6A** | IgG | ns | 1.01 | *0.0039* | 1.54 | *0.0098* | 1.55 |
|  | IgM | ns | 0.96 | *0.0024* | 1.34 | *0.0174* | 1.29 |
|  | IgA | ns | 0.73 | ns | 1.27 | ns | 0.93 |
| **HSV1** | IgG | ns | 0.23 | ns | 1.13 | ns | 0.26 |
|  | IgM | na | na | na | na | na | na |
|  | IgA | ns | 1.11 | ns | 6.99 | ns | 7.77 |
| **HSV2** | IgG | ns | 0.23 | ns | 1.04 | ns | 0.24 |
|  | IgM | ns | 0.96 | ns | 1.25 | ns | 1.21 |
|  | IgA | ns | 1.07 | ns | 1.39 | ns | 1.48 |
| **VZV** | IgG | na | na | na | na | na | na |
|  | IgM | na | na | na | na | na | na |
|  | IgA | na | na | na | na | na | na |
| **CMV** | IgG | ns | 0.81 | ns | 1.58 | ns | 1.28 |
|  | IgM | na | na | na | na | na | na |
|  | IgA | na | na | na | na | na | na |

vs: versus, ns: non-significant, na: not applicable, values that are negative

**Table S2B. Statistical significance and fold-change in saliva within the healthy donor cohort (before adjusting for age and gender)**

|  |  | **Systemic-HDs vs Local-HDs** | | **Local-HDs vs Negative-HDs** | | **Systemic-HDs vs Negative-HDs** | |
| --- | --- | --- | --- | --- | --- | --- | --- |
|  |  | ***p-value*** | **fold-change** | ***p-value*** | **fold-change** | ***p-value*** | **fold-change** |
| **SARS-CoV-2-RBD** | IgG | ns | 1.56 | *0.0022* | 4.41 | *<0.0001* | *6.89* |
|  | IgM | *0.0062* | 0.44 | *<0.0001* | 4.66 | *0.0323* | 2.05 |
|  | IgA | *0.0069* | 0.57 | *<0.0001* | 2.60 | *0.0287* | *1.49* |
| **SARS-CoV-2-NP** | IgG | ns | 3.27 | ns | 0.60 | ns | 1.98 |
|  | IgM | ns | 0.38 | ns | 2.74 | ns | 1.03 |
|  | IgA | ns | 2.54 | ns | 0.58 | ns | 1.47 |
| **HERV-K** | IgG | ns | 0.52 | *0.0329* | 2.22 | ns | 1.15 |
|  | IgM | ns | 0.78 | ns | 1.92 | ns | 1.49 |
|  | IgA | ns | 0.66 | ns | 1.70 | ns | 1.13 |
| **EBV-VCA** | IgG | ns | 0.60 | *0.0040* | 2.38 | ns | 1.42 |
|  | IgM | ns | 0.81 | *0.0163* | 1.77 | *0.0153* | *1.42* |
|  | IgA | ns | 0.90 | *0.0295* | 1.83 | *0.0295* | *1.65* |
| **EBNA1** | IgG | ns | 0.34 | ns | 4.94 | ns | 1.69 |
|  | IgM | na | na | na | na | na | na |
|  | IgA | ns | na | ns | na | ns | 0.56 |
| **HHV6A** | IgG | ns | 0.54 | *0.0408* | 1.88 | ns | 1.02 |
|  | IgM | ns | 0.89 | ns | 1.36 | ns | 1.20 |
|  | IgA | ns | 0.62 | ns | 1.78 | ns | 1.10 |
| **HSV1** | IgG | *ns* | 153.92 | ns | 0.01 | ns | 0.89 |
|  | IgM | na | na | na | na | na | na |
|  | IgA | ns | na | ns | na | ns | 27.58 |
| **HSV2** | IgG | ns | 3.19 | ns | 0.41 | ns | 1.30 |
|  | IgM | ns | 1.08 | ns | 0.93 | ns | 1.01 |
|  | IgA | ns | na | ns | na | ns | 1.61 |
| **VZV** | IgG | na | na | na | na | na | na |
|  | IgM | na | na | na | na | na | na |
|  | IgA | na | na | na | na | na | na |
| **CMV** | IgG | ns | 5.78 | ns | 0.14 | ns | 0.84 |
|  | IgM | na | na | na | na | na | na |
|  | IgA | na | na | na | na | na | na |

vs: versus, ns: non-significant, na: not applicable, values that are negative

**Table S3. Statistical significance and fold-change in saliva between the ME/CFS and HD cohorts (before adjusting for age and gender)**

|  |  | **Systemic-ME vs Systemic-HDs** | | **Local-ME vs Local-HDs** | | **Negative-ME vs Negative-HDs** | |
| --- | --- | --- | --- | --- | --- | --- | --- |
|  |  | ***p-value*** | **fold-change** | ***p-value*** | **fold-change** | ***p-value*** | **fold-change** |
| **SARS-CoV-2-RBD** | IgG | *0.0136* | 2.16 | *0.0176* | 1.68 | ns | 1.66 |
|  | IgM | *0.0264* | 1.42 | ns | 0.77 | ns | 1.14 |
|  | IgA | ns | 1.53 | ns | 0.88 | ns | 1.43 |
| **SARS-CoV-2-NP** | IgG | ns | 2.05 | ns | 1.64 | ns | 1.00 |
|  | IgM | ns | 1.52 | ns | 0.61 | ns | 0.85 |
|  | IgA | ns | 1.03 | ns | 2.29 | ns | 1.49 |
| **HERV-K** | IgG | *0.0208* | 2.60 | ns | 1.67 | ns | 1.31 |
|  | IgM | ns | 0.89 | ns | 0.70 | ns | 0.72 |
|  | IgA | ns | 0.98 | ns | 1.05 | ns | 1.16 |
| **EBV-VCA** | IgG | *0.0392* | 2.19 | ns | 1.73 | *0.0392* | 1.61 |
|  | IgM | ns | 1.05 | ns | 0.97 | ns | 0.92 |
|  | IgA | ns | 1.02 | ns | 1.35 | ns | 1.05 |
| **EBNA1** | IgG | ns | 3.30 | ns | 1.46 | ns | 1.91 |
|  | IgM | na | na | na | na | na | na |
|  | IgA | ns | 6.02 | ns | na | ns | na |
| **HHV6A** | IgG | 0.0229 | 1.53 | ns | 0.82 | ns | 1.01 |
|  | IgM | ns | 0.98 | ns | 0.90 | ns | 0.91 |
|  | IgA | ns | 0.98 | ns | 0.83 | ns | 1.16 |
| **HSV1** | IgG | ns | 1.07 | *0.0062* | 718.32 | ns | 3.69 |
|  | IgM | na | na | na | na | na | na |
|  | IgA | ns | 1.53 | ns | na | ns | 5.42 |
| **HSV2** | IgG | ns | 0.68 | *0.0307* | 9.38 | ns | 3.66 |
|  | IgM | ns | 0.93 | ns | 1.05 | ns | 0.78 |
|  | IgA | ns | 0.74 | ns | na | ns | 0.81 |
| **VZV** | IgG | na | na | na | na | na | na |
|  | IgM | na | na | na | na | na | na |
|  | IgA | na | na | na | na | na | na |
| **CMV** | IgG | ns | 2.77 | ns | 19.70 | ns | 1.81 |
|  | IgM | na | na | na | na | na | na |
|  | IgA | na | na | na | na | na | na |

vs: versus, ns: non-significant, na: not applicable, values that are negative

**Table S4A. Statistical significance in plasma within the ME/CFS cohort**

| **IgG** | **Systemic-ME vs Local-ME** | **Local-ME vs Negative-ME** | **Systemic-ME vs Negative-ME** |
| --- | --- | --- | --- |
| **SARS-CoV-2-RBD** | *<0.0001* | ns | *<0.0001* |
| **SARS-CoV-2-NP** | *<0.0001* | ns | *<0.0001* |
| **EBV-VCA** | ns | ns | ns |
| **HSV-1** | ns | ns | ns |

vs: versus, ns: non-significant, na: not applicable, values that are negative

**Table S4B. Statistical significance in plasma within the healthy donor cohort**

| **IgG** | **Systemic-HDs vs Local-HDs** | **Local-HDs vs Negative-HDs** | **Systemic-HDs vs Negative-HDs** |
| --- | --- | --- | --- |
| **SARS-CoV-2-RBD** | *<0.0001* | ns | *<0.0001* |
| **SARS-CoV-2-NP** | *0.0046* | ns | *0.0051* |
| **EBV-VCA** | ns | ns | ns |
| **HSV-1** | ns | ns | ns |

vs: versus, ns: non-significant, na: not applicable, values that are negative

**Table S5.** Statistical significance within the HDs cohort following multiple linear regression analysis taking into account age and sex as confounding factors (p values) and following two-stage linear step-up procedure of Benjamini, Krieger and Yekutieli with Q:5% (q values).

|  | **Neg-HDs vs Loc-HDs** | | **Neg-HDs vs Sys-HDs** | | **Loc-HDs vs Sys-HDs** | |
| --- | --- | --- | --- | --- | --- | --- |
|  | p value | q value | p value | q value | p value | q value |
| **CMV IgG** | 0.5276 | 0.3561 | 0.8253 | 0.9128 | 0.3139 | 0.5381 |
| **EBNA1 IgG** | 0.3527 | 0.2778 | 0.7329 | 0.9128 | 0.649 | 0.9086 |
| **HERVK IgA** | ***0.0143*** | ***0.0193*** | ***0.0473*** | 0.2649 | 0.8073 | 0.9361 |
| **HERVK IgG** | ***0.0259*** | ***0.0306*** | 0.4443 | 0.7464 | 0.1858 | 0.4459 |
| **HERVK IgM** | ***0.0007*** | ***0.0035*** | 0.1468 | 0.3828 | 0.0779 | 0.2617 |
| **HHV6A IgA** | ***0.0014*** | ***0.0035*** | 0.1147 | 0.3828 | 0.0772 | 0.2617 |
| **HHV6A IgG** | 0.4801 | 0.3490 | 0.3396 | 0.7132 | 0.3203 | 0.5381 |
| **HHV6A IgM** | ***0.0089*** | ***0.0140*** | 0.8693 | 0.9128 | 0.0652 | 0.2617 |
| **HSV1 IgA** | 0.8029 | 0.4742 | 0.4219 | 0.7464 | 0.2948 | 0.5381 |
| **HSV1 IgG** | 0.7151 | 0.4505 | 0.7846 | 0.9128 | 0.7493 | 0.9361 |
| **HSV2 IgA** | 0.1468 | 0.1541 | 0.6376 | 0.8926 | 0.0751 | 0.2617 |
| **HSV2 IgG** | 0.3164 | 0.2718 | 0.5917 | 0.8926 | 0.377 | 0.5758 |
| **HSV2 IgM** | 0.2581 | 0.2439 | 0.1595 | 0.3828 | 0.1076 | 0.3013 |
| **VCA IgA** | ***0.0015*** | ***0.0035*** | ***0.0327*** | 0.2649 | 0.0651 | 0.2617 |
| **VCA IgG** | ***0.0014*** | ***0.0035*** | 0.0634 | 0.2663 | 0.9865 | 1.0000 |
| **VCA IgM** | ***0.0071*** | ***0.0134*** | ***0.0141*** | 0.2369 | 0.8358 | 0.9361 |

**Table S6.** Statistical significance within the ME/CFS cohort following multiple linear regression analysis taking into account age and sex as confounding factors (p values) and following two-stage linear step-up procedure of Benjamini, Krieger and Yekutieli with Q:5% (q values).

|  | **Neg-ME vs Loc-ME** | | **Neg-ME vs Sys-ME** | | **Loc-ME vs Sys-ME** | |
| --- | --- | --- | --- | --- | --- | --- |
|  | p value | q value | p value | q value | p value | q value |
| **CMV IgG** | 0.6486 | 0.4994 | 0.1742 | 0.1548 | 0.3374 | 0.8666 |
| **EBNA1 IgG** | ***0.0206*** | ***0.0397*** | ***0.0046*** | ***0.0177*** | 0.7181 | 0.8666 |
| **HERVK IgA** | 0.6195 | 0.4994 | 0.4315 | 0.3323 | 0.5098 | 0.8666 |
| **HERVK IgG** | ***0.0001*** | ***0.0004*** | ***0.0001*** | ***0.0012*** | 0.2423 | 0.8666 |
| **HERVK IgM** | 0.1624 | 0.1705 | 0.1142 | 0.1099 | 0.5102 | 0.8666 |
| **HHV6A IgA** | 0.3675 | 0.3537 | 0.1087 | 0.1099 | 0.1444 | 0.8666 |
| **HHV6A IgG** | ***0.0001*** | ***0.0004*** | ***0.0131*** | ***0.0303*** | 0.4766 | 0.8666 |
| **HHV6A IgM** | ***0.0001*** | ***0.0004*** | ***0.0019*** | ***0.0110*** | 0.6176 | 0.8666 |
| **HSV1 IgA** | ***0.0309*** | 0.0510 | ***0.0439*** | 0.0845 | 0.9638 | 1.0000 |
| **HSV1 IgG** | 0.1106 | 0.1277 | 0.0703 | 0.1015 | 0.6587 | 0.8666 |
| **HSV2 IgA** | 0.7377 | 0.5325 | 0.4034 | 0.3323 | 0.251 | 0.8666 |
| **HSV2 IgG** | 0.5599 | 0.4974 | 0.5815 | 0.4198 | 0.7222 | 0.8666 |
| **HSV2 IgM** | 0.0539 | 0.0692 | 0.0983 | 0.1099 | 0.939 | 1.0000 |
| **VCA IgA** | ***0.0022*** | ***0.0051*** | 0.0545 | 0.0899 | 0.5197 | 0.8666 |
| **VCA IgG** | ***0.0018*** | ***0.0051*** | ***0.0113*** | 0***.0303*** | 0.24 | 0.8666 |
| **VCA IgM** | ***0.0465*** | 0.0671 | 0.0977 | 0.1099 | 0.4684 | 0.8666 |

**Table S7.** Statistical significance between ME/CFS and HDs following multiple linear regression analysis taking into account age and sex as confounding factors (p values) and and following two-stage linear step-up procedure of Benjamini, Krieger and Yekutieli with Q:5% (q values).

|  | **Neg-HDs vs Neg-ME** | | **Loc-HDs vs Loc-ME** | | **Sys-HDs vs Sys-ME** | |
| --- | --- | --- | --- | --- | --- | --- |
|  | p value | q value | p value | q value | p value | q value |
| **CMV IgG** | 0.1297 | 0.4086 | ***0.012*** | 0.0575 | ***0.0106*** | 0.1271 |
| **EBNA1 IgG** | 0,6004 | 0.8124 | 0.071 | 0.1529 | ***0.0227*** | 0.1271 |
| **HERVK IgA** | 0.1659 | 0.4355 | 0.5087 | 0.6574 | 0.2505 | 0.3507 |
| **HERVK IgG** | 0.7525 | 0.8124 | ***0.0171*** | 0.0575 | 0.1918 | 0.3311 |
| **HERVK IgM** | 0.8918 | 0.8779 | 0.5068 | 0.6574 | 0.233 | 0.3507 |
| **HHV6A IgA** | 0.0888 | 0.4086 | 0.9299 | 0.9764 | 0.0934 | 0.2190 |
| **HHV6A IgG** | 0.4748 | 0.7478 | ***0.0045*** | 0.0538 | ***0.0227*** | 0.1271 |
| **HHV6A IgM** | 0.4479 | 0.7478 | 0.0728 | 0.1529 | 0.1043 | 0.2190 |
| **HSV1 IgA** | 0.7508 | 0.8124 | ***0.0371*** | 0.1039 | ***0.046*** | 0.1546 |
| **HSV1 IgG** | 0.1187 | 0.4086 | ***0.0143*** | 0.0575 | ***0.0353*** | 0.1483 |
| **HSV2 IgA** | 0.1975 | 0.4444 | 0.9103 | 0.9764 | 0.2939 | 0.3798 |
| **HSV2 IgG** | 0.0592 | 0.4086 | ***0.0064*** | 0.0538 | 0.0708 | 0.1982 |
| **HSV2 IgM** | 0.3329 | 0.6554 | 0.4478 | 0.6574 | 0.5391 | 0.6038 |
| **VCA IgA** | 0.7737 | 0.8124 | 0.7283 | 0.8740 | 0.1971 | 0.3311 |
| **VCA IgG** | ***0.0001*** | ***0.0016*** | 0.1575 | 0.2940 | 0.8965 | 0.9413 |
| **VCA IgM** | 0.7352 | 0.8124 | 0.2595 | 0.4360 | 0.3974 | 0.4769 |
